# Architecting Porosity Through Monomer Engineering: Hypercrosslinked Polymers for Highly Selective CO_2_ Capture from CH_4_ or N_2_

**DOI:** 10.3390/polym17121592

**Published:** 2025-06-06

**Authors:** Lin Liu, Qi Zhang, Xue Leng, Rui Song, Zheng-Bo Han

**Affiliations:** College of Chemistry, Liaoning University, Shenyang 110036, China; liulin@lnu.edu.cn (L.L.); entropyomega@163.com (Q.Z.); xuelll99@163.com (X.L.); 17361731073@163.com (R.S.)

**Keywords:** hypercrosslinked polymers, Friedel–Crafts reaction, solvent knitting, CO_2_ purification, gas separation

## Abstract

Natural gas purification and the mitigation of carbon dioxide (CO_2_) emissions from flue gases are critical steps in alleviating the greenhouse effect and significantly mitigate multiple environmental challenges associated with global warming. Hypercrosslinked polymers (HCPs) have become a hot topic as prospective adsorbents for gas purification and separation, owing to their low cost and scalability. Hence, TPB-Ben, TPB-Nap, and TPB-Ant were synthesized through a solvent knitting strategy, with the modification in the size of the monomers serving as a distinctive feature. This alteration aimed to explore the impact of phenyl ring quantity on the polymers’ gas adsorption and separation efficiency. All HCPs showed outstanding selective separation capability of CO_2_ from CO_2_/CH_4_ and CO_2_/N_2_ mixtures, such as TPB-Ben-3-2 (CO_2_/CH_4_: 10.77; CO_2_/N_2_: 59.72), TPB-Nap-3-2 (CO_2_/CH_4_: 9.12; CO_2_/N_2_: 61.31), and TPB-Ant-3-2 (CO_2_/CH_4_: 10.00; CO_2_/N_2_: 62.89), which could be potential candidate adsorbents for natural gas purification and CO_2_ capture. Considering the mild reaction conditions, low cost, efficient gas adsorption, and the potential for scalable production, these polymers are considered ideal selective solid adsorbents for capturing CO_2_. This further highlights the significance of the solvent knitting strategy.

## 1. Introduction

As societal demands stemming from production and daily life continue to expand, the corresponding need for energy has witnessed a parallel increase [1]. Carbon dioxide (CO_2_), a gas constituting the major fraction of greenhouse gas (GHG), is a major secondary product from fossil fuel combustion, where flue gas led by coal and petroleum is a complex composition with about 85% N_2_ and 15% CO_2_ [2]. As a result, excessive emissions cause the increasing concentration of CO_2_ in the atmosphere [3,4,5], leading to serious environmental issues, including global warming [6,7,8], ocean acidification, extreme weather [9], and species extinction. Additionally, natural gas (NG), as one of the cleanest fossil fuels, consists primarily of CH_4_, CO_2_, and light hydrocarbons. Natural gas utilization heavily relies on pipeline transportation, and the CO_2_ impurity in natural gas can significantly reduce its energy density and severely damage gas pipelines [10]. Therefore, developing the technology of capturing and separating CO_2_ from flue gas and natural gas is highly promising for mitigating CO_2_ emissions and enhancing natural gas quality. 

In recent years, capturing CO_2_ with porous materials as adsorbent has been considered as an efficient, convenient, and economical technology to replace the traditional liquid amine adsorbents. A diverse range of porous sorbent materials, including Metal-Organic frameworks (MOFs), zeolites, and activated carbons, have been extensively investigated for carbon capture and storage (CCS) applications. Among them, Hypercrosslinked Polymers (HCPs), which represent a subclass of microporous organic polymers, have received increasing attention for their capability to absorb CO_2_, and they demonstrate the enormous potential for various applications in gas capture and storage, drug delivery, chromatographic separations, super capacitors, water treatment, and molecular separation, etc. [11,12,13]. HCPs can be synthesized through simple methods, are low in cost, and are easy to produce in large quantities. HCPs are readily prepared through mild Friedel–Crafts alkylation reaction, allowing scalable synthesis from low-cost precursors [14]. The highly crosslinked nature provides HCPs’ high surface area, good thermal properties, high reaction yield, and excellent chemical stability [15,16]. These characteristics make HCPs excellent gas adsorbent candidates for clean energy and environmental applications, compensating the disadvantages such as physicochemical instability, costly and toxic starting monomers, expensive catalysts, harsh synthetic conditions, and so on.

HCPs are usually synthesized by crosslinking two monomers through Friedel–Crafts reactions [17]. In recent years, HCPs are developed via three primary routes [18,19,20]: (1) post-crosslinking of polymer precursors; (2) one-step polycondensation of monomers; or (3) hypercrosslinking of aromatics using external linkers. With the deep understanding of HCPs, the synthesis method relying on the solvent knitting strategy has been progressively developed, but there has been a notable scarcity of studies focusing on its application in gas separation and CO_2_ capture. Bien Tan et al. reported that materials synthesized via the solvent knitting method, which exhibited higher CO_2_ capture ability than the HCPs synthesized via the knitting method with formaldehyde dimethyl acetal (FDA) or the Scholl coupling reaction [14]. Based on various amine building blocks, Tan et al. made three novel polymers synthesized via the solvent knitting method [21]. According to the reported literature, phenyl-rich HCPs exhibited higher affinity due to π-electron/quadrupole interactions with CO_2_ (quadrupolar), rather than the non-polar CH_4_ molecules (small octupole moment) or N_2_ molecules. This difference in affinity enhanced the material’s ability to selectively adsorb CO_2_ from CH_4_ or N_2_, thus accomplished the effective separation of the CO_2_/CH_4_ and CO_2_/N_2_ mixture.

Here, a series of hypercrosslinked polymers (HCPs), denoted as TPB-Ben, TPB-Nap, and TPB-Ant, were successfully synthesized via a solvent knitting strategy by copolymerizing a twisted and rigid 1,3,5-triphenylbenzene (TPB) monomer with aromatic comonomers of progressively increasing sizes (benzene, naphthalene, and anthracene). This systematic monomer size variation enabled precise regulation of the polymer pore architectures, resulting in tunable pore size distributions and enhanced gas adsorption capacities, particularly for CO_2_ capture applications. By analyzing the data of CO_2_ single-component gas adsorption experiments of HCPs synthesized at various ratios and monomers, it was clarified that the difference in their CO_2_ adsorption capacity is caused by different proportions and monomers. Subsequently, the materials’ practical separation capabilities were ascertained by dynamic breakthrough experiments with CO_2_/CH_4_ and CO_2_/N_2_ mixed gases. Meanwhile, the strategy proposes a method for enhancing CO₂ adsorption performance by regulating the interaction between molecules and the adsorbent surface through the selection of appropriate monomers. Specifically, this approach achieves the optimization of adsorption capacity by deliberately choosing monomers that can modulate the strength and nature of molecular interactions with the adsorbent surface, thereby improving the material’s affinity and selectivity for CO_2_ molecules. Generally, this work provided a design approach for the practical industrial applications of the solvent knitting strategy and initiated the new possibilities to prepare HCPs with gas adsorption and separation for targeted applications.

## 2. Experimental Section

### 2.1. Materials

All commercial-grade reagents were used without further treatment. 1,3,5-triphenylbenzene (TPB, 99%+ purity) was purchased from the Explore platform and can be used directly without any further purification. Benzene, anhydrous aluminum chloride (AlCl_3_), and hydrochloric acid (HCl) were obtained from China National Pharmaceutical Group Corporation, Beijing, China. Naphthalene and anthracene were sourced from Tianjin Yongda Chemical Reagent Co., Ltd., Tianjin, China. 1,2-dichloroethane (DCE), dichloromethane, and ethanol from Tianjin Fuyu Fine Chemical Co., Ltd., Tianjin, China.

### 2.2. Instrumentation

Powder X-ray diffraction (PXRD) patterns were acquired by using a Bruker AXS D8 system (Bruker AXS GmbH, Karlsruhe, Germany) with Cu-Kα radiation (λ = 1.5406 Å) and the scans were performed over a range of 5–50° at 40 kV and 40 mA. Fourier Transform Infrared (FT-IR) spectroscopy was conducted using a Nicolet 5DX spectrometer (Thermo Fisher Scientific Inc., Waltham, MA, USA). Thermogravimetric analysis (TGA) was conducted using a Precision RZY-1 analyzer (instruments and electronics (Shanghai) associates, Shanghai, China) under a nitrogen atmosphere (10 mL/min), with the temperature ramped from ambient to 800 °C at a rate of 10 °C/min. To characterize the pore structure and surface properties of the materials, N_2_ adsorption–desorption isotherms were measured at 77 K using a BeiShiDe 3H-2000PS2 system (Beishide Instrument Technology (Beijing) Co., Ltd., Beijing, China), which was employed to analyze the textural features of the materials through gas adsorption behavior at cryogenic temperatures. Before testing, the sample was placed under vacuum and heated at 110 °C for 12 h to remove guest molecules. The morphology of the polymer was investigated by using Hitachi SU-8010 (Hitachi, Ltd., Tokyo, Japan) at 5.0 kV at 2.5–5 k magnification. ^13^C Cross-Polarization/Magic Angle Spinning Nuclear Magnetic Resonance (^13^C CP/MAS NMR) tests were conducted on Bruker AVANCE NEO 400WB, Karlsruhe, Germany.

### 2.3. Expertmental Methods

Prior to single-component gas adsorption measurements, all samples were de-gassed at 110 °C under vacuum for 12 h to remove the solvent. Nitrogen (N_2_) adsorption isotherms were then collected at 77 K using a 3H-2000PS2 analyzer. The Brunauer–Emmett–Teller (BET) surface area and pore size distribution were calculated from the test results of 77 K N_2_ adsorption satisfying the standard of the Rouquerol consistency criteria. The single-component adsorption measurements of CO_2_, N_2_, and CH_4_ were then tested at 273 K and 298 K, which are two temperatures commonly adopted in gas separation field research. 

The dynamic breakthrough experiments were carried out in a unique device designed by Nanjing Haoerpu Analytical Equipment Co., Ltd., Nanjing, China. Before starting the test, all samples need to be activated, as described previously. The samples, including TPB-Ben-3-2, TPB-Nap-3-2, and TPB-Ant-3-2, were loaded into a stainless-steel tube with 200 mm length and 4 mm inner diameter. The sample loading amount was 1 g each time, and only one type of sample was loaded. After being loaded onto the device, the stainless-steel tube was heated to 353 K under vacuum overnight. Pure single-component gases were mixed to a fixed ratio (CO_2_/CH_4_, 50/50 *v/v*; 15/85 *v/v*; CO_2_/N_2_, 50/50 *v/v*; 15/85 *v/v*) in the device for breakthrough experiments. Subsequently, the breakthrough experiments were conducted at 1 bar at 273 K with a flow rate of 2 mL/min of the mixed gas. All breakthrough measurements utilized fixed operational parameters, while real-time chromatographic analysis characterized the effluent gas mixture. After the test was completed, the sample was eluted with He gas flow (10 mL/min) at 353 K for a period of 30 min and prepared for the next test. 

### 2.4. Synthesis Methods

TPB-Ben-3-1 was synthesized by a solvent knitting strategy [14] through the Friedel–Crafts reaction. 1,3,5-triphenylbenzene (TPB) (3 mmol, 0.92 g) and benzene (1 mmol, 0.089 mL) were dissolved in 40 mL of 1,2-dichloroethane (DCE) under nitrogen atmosphere and stirred for 30 minutes until a clear solution was formed. Subsequently, 80 mmol (10.67 g) of anhydrous AlCl_3_ was introduced into the solution. The reaction system was then stirred at gradually increasing temperature (20 °C, 4 h; 30 °C, 8 h; 40 °C, 12 h; 60 °C, 12 h; 80 °C, 36 h). Following cooling to 25 °C, the reaction was quenched with 100 mL of HCl-H2O (*v/v* = 2:1), and the synthesized black powder was washed several times by means of deionized water, dichloroethane, anhydrous ethanol, and dichloromethane. Finally, Soxhlet extraction was performed in ethanol for 48 h, and the extract was dried in a vacuum oven for 24 h at 70 °C (yield, 149%). According to the general synthesis procedure, a series of TPB-Ben materials with different molar ratios were prepared by maintaining a constant molar amount of 1,3,5-triphenylbenzene (TPB) while adjusting the dosage of benzene in the copolymerization system. Specifically, through systematically varying the content of benzene relative to the fixed TPB amount, TPB-Ben materials with diverse compositional ratios were successfully synthesized in this copolymerization process.

Following the synthesis conditions of TPB-Ben-3-1, TPB-Nap-3-1 was produced by treating naphthalene (1 mmol, 0.1282 g) with AlCl_3_ (80 mmol, 10.67 g) in DCE (40 mL) (yield, 169%). Along with the general synthesis procedure, a range of TPB-Nap materials with different molar ratios were synthesized by keeping the molar amount of 1,3,5-triphenylbenzene (TPB) constant while altering the quantity of naphthalene in the copolymerization system. Specifically, through systematically adjusting the dosage of naphthalene relative to the fixed TPB molar ratio, TPB-Nap materials with diverse compositional proportions were successfully prepared in this copolymerization process.

Following the synthesis conditions of TPB-Ben-3-1, TPB-Ant-3-1 was produced by treating anthracene (1 mmol, 0.178 g) with AlCl_3_ (80 mmol, 10.67 g) in DCE (40 mL) (yield, 145%). Following the general synthesis procedure, a series of TPB-Ant materials with distinct molar ratios were prepared by maintaining a constant molar amount of 1,3,5-triphenylbenzene (TPB) while systematically varying the dosage of anthracene in the copolymerization system. Through this approach, TPB-Ant materials with diverse compositional ratios were successfully synthesized, enabling precise control over the molar proportion of anthracene relative to the fixed TPB content in the copolymerization process.

## 3. Results and Discussion

### 3.1. Structural Characterization

**Scheme 1:**
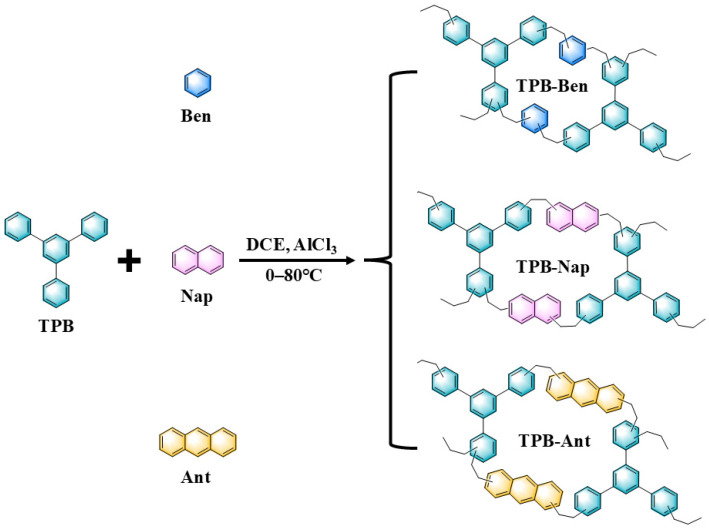
Schematic illustration of TPB-Ben-x-y, TPB-Nap-x-y, and TPB-Ant-x-y synthesized by the solvent knitting strategy.

Based on the Friedel–Crafts reaction and employing the solvent knitting strategy, a diverse series of microporous Hypercrosslinked Polymers (HCPs) featuring different monomer sizes were synthesized. By copolymerizing 1,3,5-triphenylbenzene (TPB) with benzene (Ben), naphthalene (Nap), or anthracene (Ant) at varying molar ratios (x/y), three polymer families (TPB-Ben-x-y, TPB-Nap-x-y, TPB-Ant-x-y) with tailored porosity were obtained. Scheme 1 illustrates the general synthetic route for HCPs. The differences in monomer proportions and sizes lead to distinct pore environments, resulting in varied gas adsorption performances.

**Figure 1:**
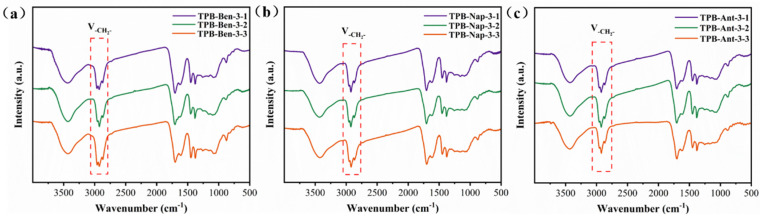
FT-IR spectra of (**a**) TPB-Ben, (**b**) TPB-Nap, and (**c**) TPB-Ant showing the appearance of characteristic peaks of C-H bonds in hypercrosslinked polymers.

The successful formation of TPB-Ben, TPB-Nap, and TPB-Ant was corroborated by Fourier transform infrared (FT-IR) spectroscopy (Figure 1), which verified the structure of hypercrosslinked polymers. Due to the occurrence of the Friedel–Crafts alkylation reaction, the peak located on 2926 cm^−1^ in HCPs could be ascribed to the stretching vibrations of the methylene group of FDA. Three diagnostic bands (1700, 1600, and 1450 cm^–1^) in the fingerprint region correspond to the aromatic skeleton vibrations, matching hypercrosslinked polymer signatures [12]. The appearance of the same peaks clearly confirmed the successful synthesis of the HCPs. The results of PXRD testing revealed their amorphous structure, which could be proven by the broad peaks presented in the curves (Figure S1). All the HCPs exhibited a wide peak, further confirming the success of the knitting reaction. Thermogravimetric analysis (TGA) was conducted to verify the stability of the samples as illustrated in Figure S2. All these materials exhibited good thermal stability with a mass loss till 550 K, which verified that all the HCPs exhibit good thermal stability. It was helpful to maintain its performance over multiple adsorption/desorption cycles and practical applications.

**Figure 2:**
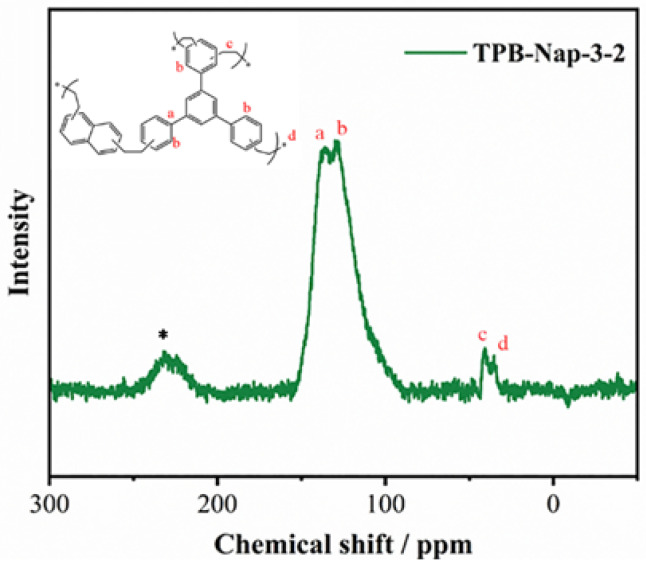
Solid-state ^13^C NMR spectra of TPB-Nap-3-2 exhibiting characteristic resonance for aromatic (substituted and unsubstituted) and bridging methylene carbons. The letters and symbols located on the peaks in the figure correspond to the C atoms at the respective positions.

Solid state ^13^C CP/MAS NMR revealed the structural of TPB-Nap-3-2 as a representative (Figure 2). The peaks at 130 ppm were attributed to the aromatic carbon without substitution and peaks emerged near 137 ppm belonging to the aromatic carbon with substitution. More importantly, the formation of the HCP framework was evidenced by the diagnostic methylene carbon signal (around 37 ppm). Given the FT-IR and ^13^C CP/MAS NMR results, microporous HCPs were successfully synthesized by the simple solvent knitting method. Additionally, the morphology of the hypercrosslinked polymers (TPB-Ben-3-2, TPB-Nap-3-2, TPB-Ant-3-2) was observed by using SEM. The SEM micrographs demonstrate an amorphous state, which is like the reported HCPs, especially those synthesized by the solvent knitting strategy. This confirmed the existence of amorphous and porous morphological structures in these polymers (Figure S3) [14].

**Figure 3:**
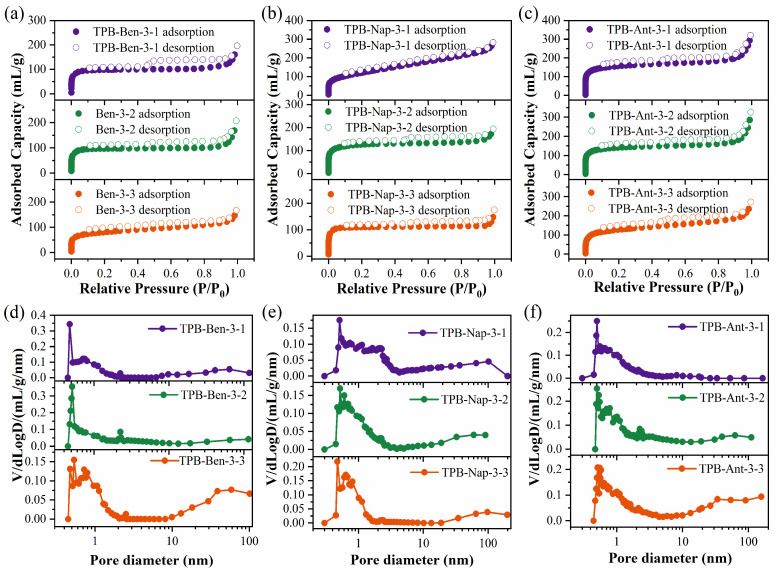
Adsorption and desorption isotherms of N_2_ at 77 K: (**a**) TPB-Ben, (**b**) TPB-Nap, (**c**) TPB-Ant, and aperture distribution: (**d**) TPB-Ben, (**e**) TPB-Nap, (**f**) TPB-Ant reflecting abundant micropore structure and macropore formation.

The N_2_ adsorption analysis at 77 K were used to investigate the pore structure and pore size distribution of TPB-Ben, TPB-Nap, and TPB-Ant. The isotherms of TPB-Ben, TPB-Nap, and TPB-Ant exhibited a type I adsorption isotherm. As shown in Figure 3, the adsorption isotherms of all samples reflected abundant micropore structure and the macropores formation [22]. Pore size distribution was calculated from the results of the N_2_ adsorption through nonlocal density functional theory (NLDFT). All synthesized HCPs’ pore size distributions are shown in Figure 3d–f. The Brunauer–Emmett–Teller (BET) surface area gradually increased with the addition of benzene ring numbers, and the monomer ratio also influenced the change in the BET surface. Furthermore, because of their high porosity and robust C-C bonds, the materials remained stable even in wet operating conditions [15]. When further enhancing the molar ratio of monomer benzene, naphthalene, or anthracene (3:1, 3:2, and 3:3), the BET surface area exhibits a declining trend in Figure 3. This phenomenon indicates successful copolymerization, where pore blockage occurring leads to a reduction in the BET surface area, such that the BET surface areas of TPB-Ben-3-3, TPB-Nap-3-3, and TPB-Ant-3-3 are 268, 335, and 420 m^2^/g, respectively. The decline in the BET surface area of HCPs may be attributed to two reasons. One reason is the reduced free packing density in the polymer structure at elevated monomer concentrations, and the other is the reduction in volume, which limits the access of N_2_ or CH_4_ into the narrow pores [23]. By comparing the specific surface and micropore size, it was found that an appropriate monomer ratio can make HCPs have more suitable micropores and a larger specific surface area. Therefore, a pore structure more suitable for gas separation can be obtained by changing the proportion of monomers in the solvent knitting method.

### 3.2. Single-Component Gas Adsorption Tests

**Figure 4:**
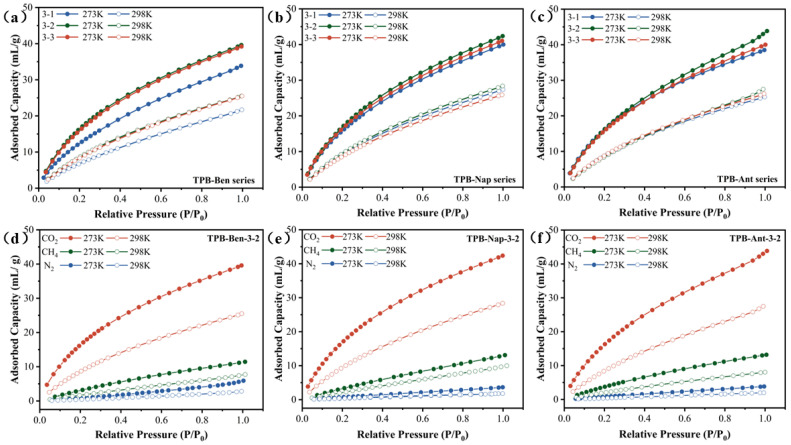
Adsorption isotherms of CO_2_ in (**a**) TPB-Ben, (**b**) TPB-Nap-3-2, (**c**) TPB-Ant-3-2. Adsorption isotherms of CO_2_, CH_4_, and N_2_ in (**d**) TPB-Ben-3-2, (**e**) TPB-Nap-3-2, (**f**) TPB-Ant-3-2 demonstrating its gas separation potential.

Given the microporous architecture and large surface area of synthesized HCPs, single-component gas adsorption tests of CO_2_, CH_4_, and N_2_ were conducted to investigate their gas uptake capacities. Experiments with single-component gases were conducted to demonstrate the adsorption capability of the synthesized HCPs. Based on the CO_2_ isotherms of polymers, the optimal molar ratio for the reaction was established. TPB-Ben-3-2, TPB-Nap-3-2, and TPB-Ant-3-2 exhibited better CO_2_ uptake capacity (where the molar ratio of 1,3,5-triphenylbenzene to monomer is 3:2). Figure 4 shows the CO_2_, N_2_, and CH_4_ adsorption capacity of the HCPs at 273 K and 298 K. The CO_2_ adsorption capacities of TPB-Ben-3-2, TPB-Nap-3-2, and TPB-Ant-3-2 were 39.6, 42.4, and 43.9 mL/g at 273 K, respectively. Compared with CH_4_ and N_2_, these HCPs exhibited a higher absorption capacity for CO_2_. In comparison, the CH_4_ adsorption capacity (11.4, 13.1, and 13.2 mL/g at 273 K) is significantly lower than that of CO_2_. Additionally, the N_2_ adsorption amounts (5.9, 3.7, and 3.8 mL/g at 273 K) are nearly negligible.

**Figure 5:**
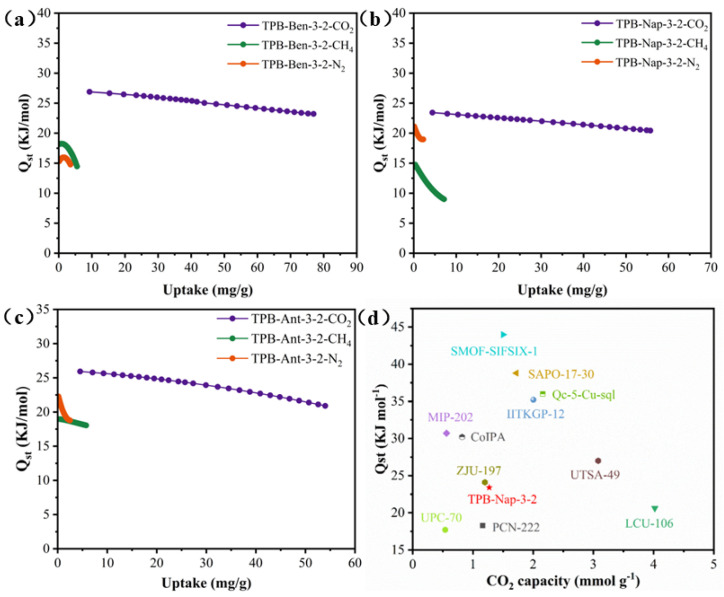
Isosteric heat of adsorption of CO_2_, CH_4_, and N_2_ in (**a**) TPB-Ben-3-2, (**b**) TPB-Nap-3-2, (**c**) TPB-Ant-3-2. (**d**) Comparison of CO_2_ adsorption capacity and *Q*_st_ values with reported porous materials demonstrating superior separation capability.

The isosteric heat of adsorption (*Q*_st_) was computed by using the Clapeyron returns according to the results of single-component gas adsorption at 273 K and 298 K, which was fitted by the virial equation (Figures S4–S12) [24]. As shown in Figure 5, the *Q*_st_ for CO_2_ was 20.4–23.4 kJ/mol in TPB-Nap-3-2; the moderate *Q*_st_ facilitated CO_2_ desorption from the framework, demonstrating outstanding regeneration efficiency in the practical separation process. The *Q*_st_ for CO_2_ was 23.2–26.9 kJ/mol in TPB-Ben-3-2, and the *Q*_st_ for CO_2_ was 20.9–25.9 kJ/mol in TPB-Ant-3-2. The calculated *Q*_st_ levels of these HCPs were CO_2_ > CH_4_ > N_2_, which agreed with the order of single-gas adsorption isotherm data. It shows that HCPs have the potential to separate CO_2_/CH_4_ and CO_2_/N_2_ mixed gases. It is worth noting that a moderate amount of CO_2_ adsorption heat will reduce the difficulty of the desorption process and enhance the material’s cyclic capacity [25].

Figure 5d and Table S1 show a comparison of TPB-Nap-3-2 with other representative porous materials with respect to CO_2_ adsorption capacity and *Q*_st_ (CO_2_) values [26,27,28,29,30,31,32,33,34]. TPB-Nap-3-2 not only showed excellent CO_2_ uptake but also maintained moderate *Q*_st_, which ensured the subsequent cyclic regeneration ability of the TPB-Nap-3-2 adsorbent [35].

**Figure 6:**
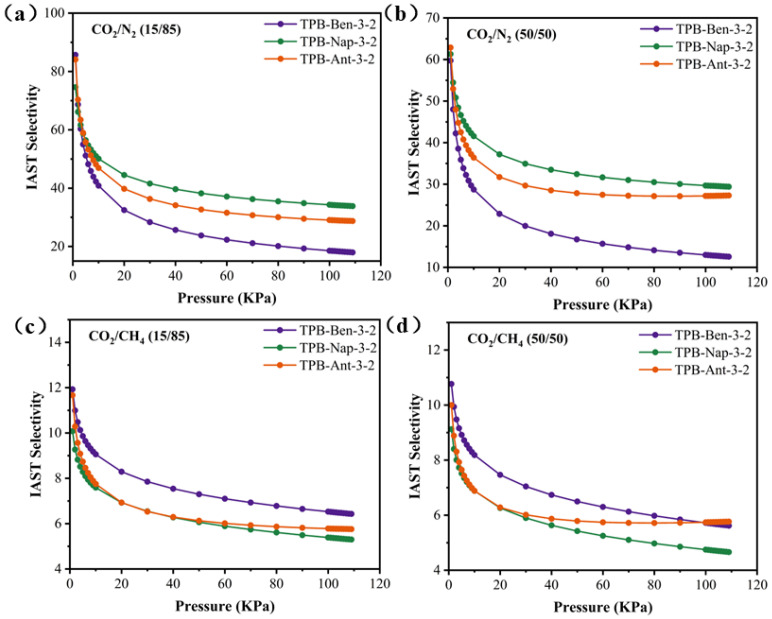
IAST selectivities for TPB-Ben-3-2, TPB-Nap-3-2, and TPB-Ant-3-2 at 298 K and above 0–1 bar: (**a**) 15/85 CO_2_/N_2_, (**b**) 50/50 CO_2_/N_2_, (**c**) 15/85 CO_2_/CH_4_, (**d**) 50/50 CO_2_/CH_4_ exhibiting high gas selectivity.

Because gas selectivity is an important criterion for evaluating the capability of adsorbents, the isotherm was fitted by using the dual-site Langmuir–Freundlich (DSLF) model (Figures S13–S15) and then we predicted the selectivity for CO_2_/CH_4_ and CO_2_/N_2_ by the ideal adsorbed solution theory (IAST) [36]. Compared with CH_4_ and N_2_, TPB-Ben-3-2, TPB-Nap-3-2, and TPB-Ant-3-2 exhibited higher absorption capacity for CO_2_. Figure 6 shows the separation selectivity of TPB-Ben-3-2, TPB-Nap-3-2, and TPB-Ant-3-2 for mixed gases with different molar ratios. Table 1 provides more detailed information on the adsorption capacity and selectivity. These materials exhibited a higher selectivity for CO_2_/N_2_ compared to CO_2_/CH_4_. Additionally, TPB-Nap-3-2 exhibited lower selectivity than TPB-Ben-3-2 and TPB-Ant-3-2, mainly because the smaller pore size of TPB-Nap-3-2 provides stronger host–guest interactions with CH_4_ and N_2_ [37].

### 3.3. Dynamic Breakthrough Experiments

**Figure 7:**
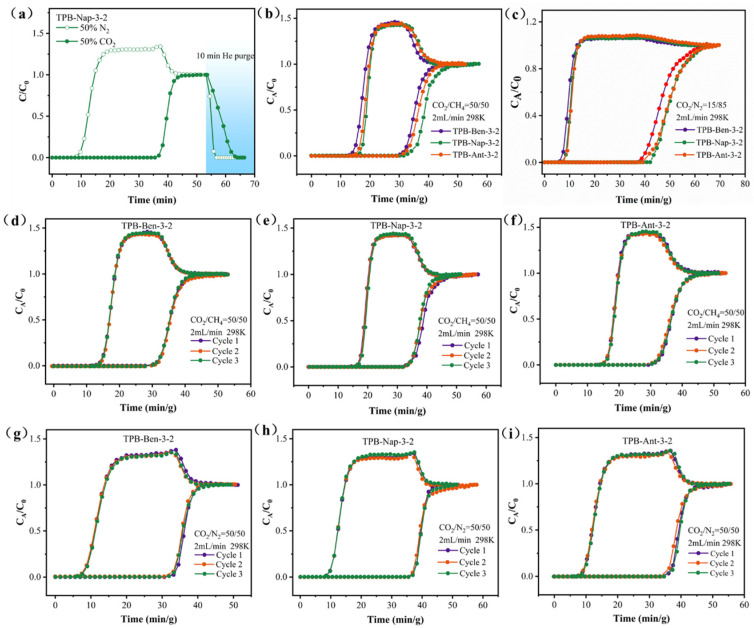
Dynamic experimental breakthrough results for (**a**) CO_2_/N_2_ (50/50) mixture in TPB-Nap-3-2 with desorption process; (**b**) CO_2_/CH_4_ (50/50) mixture in three HCPs; (**c**) CO_2_/N_2_ (15/85) mixture in three HCPs. The results of three cycles of breakthrough experiments of binary mixtures of CO_2_/CH_4_ (50/50, *v/v*) for (**d**) TPB-Ben-3-2, (**e**) TPB-Nap-3-2, and (**f**) TPB-Ant-3-2 at 298 K. The results of three cycles of breakthrough experiments of binary mixtures of CO_2_/N_2_ (50/50, *v/v*) for (**g**) TPB-Ben-3-2, (**h**) TPB-Nap-3-2, and (**i**) TPB-Ant-3-2 at 298 K validating their practical application potential.

A series of dynamic breakthrough experiments were conducted under simulated industrial production conditions to evaluate the potential for practical CO_2_ capture from flue gas. As shown in Figure 7, all the three HCPs achieved convincing CO_2_/CH_4_ and CO_2_/N_2_ separation. After comparing the separation effects of three materials at two kinds of mixed gases (CO_2_/CH_4_ and CO_2_/N_2_), we found that TPB-Nap-3-2 exhibits excellent separation performance. It was shown that CH_4_ eluted after 12 min/g, whereas CO_2_ breakthrough did not occur until 34 min, which confirmed a clear separation in TPB-Nap-3-2. This means CO_2_ was absorbed by the adsorbate and retained in the packed column for a considerable duration until it reached the state of saturated adsorption and breakthrough. The time for CO_2_ remaining was 22 min/g between CO_2_ and N_2_. The shorter breakthrough time of CH_4_, compared to CO_2_, corresponds well with the lower CH_4_ uptake from the adsorption isotherms. For the breakthrough experiment of CO_2_/N_2_ (15:85 *v*/*v*) mixture, the maximum breakthrough time for TPB-Nap-3-2 reached 36 min/g. Markedly, Figure 7a shows that the column with CO_2_ saturated materials could be rapidly regenerated with He gas flushing at a flow rate of 2 mL/min, which could be explained by its moderate *Q*_st_ (CO_2_) value (20.4–23.4 kJ/mol) of TPB-Nap-3-2.

Cyclic breakthrough experiments were executed to explore the renewability and reusability of the material. As displayed in Figure 7d–f, the breakthrough performance of the three HCPs, TPB-Ben-3-2, TPB-Nap-3-2, and TPB-Ant-3-2, remained unchanged after three recycling experiments. More importantly, after going through three cycles, the adsorbent still maintains a stable CO_2_ capture capacity. This confirmed the high stability and reusability of synthesized HCPs [38]. It means that the adsorption capacity of the HCPs is well retained after dynamic capturing, which could be a potential candidate for separation of CO_2_/CH_4_ and CO_2_/N_2_ mixtures. For CO_2_/N_2_ (15/85, *v*/*v*) binary gas mixture, TPB-Nap-3-2 afford a time interval of 36 min/g; for CO_2_/CH_4_ (50/50, *v*/*v*) binary gas mixture, TPB-Nap-3-2 afford a time interval of 20 min/g. All the results verified the separation efficiency in simulated flue gas (CO_2_/N_2_: *v*/*v* = 15/85) and natural gas (CO_2_/CH_4_: *v*/*v* = 50/50). In brief, high CO_2_ capacity and excellent recycling capacity render the HCPs excellent candidates of promising adsorbents for CO_2_ capture from flue gas and natural gas.

To further explore the adsorption mechanism for TPB-Nap-3-2, density functional theory (DFT) calculations were performed to demonstrate the interactions between the gas and the adsorbent. Considering the polymerization process, we focused on the complexes of monomers and one gas molecule to more clearly interpret the interaction relationship. The method is similar to approaches in the reported literature; host–guest affinity was evaluated by probing the structural compatibility of functional moieties with gas molecules [39,40]. DFT calculations revealed that the gas molecules preferentially were adsorbed near the benzene ring (Figure S16). The calculated static binding energy of CO_2_ in TPB-Nap-3-2 is 18.5 kJ/mol.

In short, the three HCPs all exhibited good separation performance, which is attributed not only to the conjugative interactions between the benzene rings and CO_2_ gas molecules but also to the fine tuning of monomer size. With the increase in monomer size, the porosity and specific surface of the polymers are enhanced, which provides more adsorption sites for CO_2_ molecules. Moreover, the presence of benzene rings also strengthens the polymers’ conjugated system, allowing CO_2_ molecules to be effectively adsorbed through interactions with the π electron clouds of the benzene rings (π-π interactions). However, the size of the monomers in the HCPs directly impacts their CO_2_ adsorption performance. Through comparison, we found that TPB-Nap-3-2 has the longest separation time among the three materials, demonstrating good separation performance.

## 4. Conclusions

In summary, we report a strategy for architecting porosity through monomer engineering by the facile solvent knitting and obtained TPB-Ben, TPB-Nap, and TPB-Ant. The synthesized HCPs can effectively separate CO_2_/CH_4_ mixtures, and capture CO_2_ from CO_2_/N_2_ mixtures. Notably, materials TPB-Ben-3-2, TPB-Nap-3-2, and TPB-Ant-3-2 exhibited high CO_2_/N_2_ (15/85: *v/v*) selectivity of 85.68, 74.64, and 84.10, respectively. Due to the different pore environment, the gas separation performance of HCPs with similar morphology is different. The results of the breakthrough experiments confirmed that TPB-Nap-3-2 exhibits superior separation performance of CO_2_/CH_4_ (20 min/g) and CO_2_/N_2_ (36 min/g) compared to TPB-Ben-3-2 and TPB-Ant-3-2. The stability, regenerability, and cycling capacity of the synthesized HCPs were verified through cyclic breakthrough experiments, which makes them excellent candidates for gas separation and CO_2_ capture. This work architected porosity through monomer engineering to synthesize a series of HCPs, which establishes a strategy toward designing promising adsorbents with high CO_2_ capacity. Moreover, this work also contributes to broadening the industrial application scope of HCPs.

## Figures and Tables

**Table 1:** IAST selectivity of the three best-performing HCPs at 298 K.

Adsorbent	IAST Selectivity (298 K, 1 atm)			
	CO_2_/CH_4_ (15/85)	CO_2_/CH_4_ (50/50)	CO_2_/N_2_ (15/85)	CO_2_/N_2_ (50/50)
TPB-Ben-3-2	11.93	10.77	85.68	59.72
TPB-Nap-3-2	10.08	9.12	74.64	61.31
TPB-Ant-3-2	11.67	10.00	84.10	62.89

## Data Availability

Data are contained within the article and Supplementary Materials.

## Supplementary Materials

The following supporting information can be downloaded at: https://www.mdpi.com/article/10.3390/polym17121592/s1, Figure S1. PXRD pattern of (a) TPB-Ben-3-2 (b) TPB-Nap-3-2 (c) TPB-Ant-3-2; Figure S2. The TGA curves of (a) TPB-Ben-3-2 (b) TPB-Nap-3-2 (c) TPB-Ant-3-2; Figure S3. SEM images of (a) TPB-Ben-3-2, (b) TPB-Nap-3-2, (c,d) TPB-Ant-3-2; Figure S4. The virial fitting of CO_2_ sorption data for TPB-Ben-3-2; Figure S5. The virial fitting of CH_4_ sorption data for TPB-Ben-3-2; Figure S6. The virial fitting of N_2_ sorption data for TPB-Ben-3-2; Figure S7. The virial fitting of CO_2_ sorption data for TPB-Nap-3-2; Figure S8. The virial fitting of CH_4_ sorption data for TPB-Nap-3-2; Figure S9. The virial fitting of N_2_ sorption data for TPB-Nap-3-2; Figure S10. The virial fitting of CO_2_ sorption data for TPB-Ant-3-2; Figure S11. The virial fitting of CH_4_ sorption data for TPB-Ant-3-2; Figure S12. The virial fitting of N_2_ sorption data for TPB-Ant-3-2; Figure S13. CO_2_, CH_4_, and N_2_ adsorption data of TPB-Ben-3-2 fitted by the dual site Langmuir Freundlich model at 298 K; Figure S14. CO_2_, CH_4_, and N_2_ adsorption data of TPB-Nap-3-2 fitted by the dual site Langmuir Freundlich model at 298 K; Figure S15. CO_2_, CH_4_, and N_2_ adsorption data of TPB-Ant-3-2 fitted by the dual site Langmuir Freundlich model at 298 K; Figure S16 The optimal binding sites of CO_2_ was calculated by density functional theory (O, red; C, gray). Table S1. CO_2_ adsorption capacity and *Q*_st_ values comparison with reported porous materials. References [41,42,43,44,45] are cited in the Supplementary Materials.
