# Supplementary material for: Architecting Porosity Through Monomer Engineering: Hypercrosslinked Polymers for Highly Selective CO2 Capture from CH4 or N2
Source: Polymers (Basel). 2025 Jun 6;17(12):1592. doi: 10.3390/polym17121592 (PMC12197289; doi:10.3390/polym17121592)
Supplement: Supplementary file 1 [file polymers-17-01592-s001.zip › polymers-3668056-supplementary.pdf]

Supporting Information

Architecting Porosity through Monomer Engineering: Hyper-crosslinked Polymers for Highly Selective CO_2 Capture from CH_4 or N_2

Lin Liu, Qi Zhang, Xue Leng, Rui Song, Zheng-Bo Han*

College of Chemistry, Liaoning University, Shenyang 110036 P. R. China

**Corresponding authors E-mail: ceshzb@lnu.edu.cn.*

1. Theoretical calculation method

In this study, the Single-site Langmuir-Freundlich (SSLF) model was used to correctly compute the IAST [1–5]:

$$N = A_1 \frac{b_1 p^{c_1}}{1+b_1 p^{c_1}} \quad \text{(S1)}$$

where N denotes gas uptake (mmol/g); A_1 represent the theoretical maximum adsorption capacity (mmol/g) at the 298 K; b_1 denote the correlation coefficients; $c_1$denote the site anisotropism.

The IAST selectivity was calculated as follows:

$$S_{ij} = \frac{n_i/n_j}{y_i/y_j} \quad \text{(S2)}$$

In this scenario, i and j represent the two relative gases; n_i and n_j represent gas uptake; y_i and y_j represent the gas mixture's suggested mole fractions.

Based on the adsorption isotherms of pure gas at 298 K and 273 K, Using the Clausius-Clapeyron equation, the Isosteric Heat of Adsorption (Q_{st}) were computed as follows:

$$\ln P = \ln N + \frac{1}{T}\sum_{i=0}^{m} a_i N^i + \sum_{i=0}^{n} b_i N^i \quad \text{(S3)}$$

where P denotes the pressure; N gives the amount of uptake; T refers to the temperature and m and n connote the number of words necessary to fully explain the isotherm.

$$Q_{st} = -R \sum_{i=0}^{m} a_i N^i \quad \text{(S4)}$$

Here R means the universal gas constant. The coverage dependence of the Q_{st} values was derived by fitting the adsorption data at various temperatures for TPB-Ben-3-2, TPB-Nap-3-2 and TPB-Ant-3-2.

2. Supplement Figures

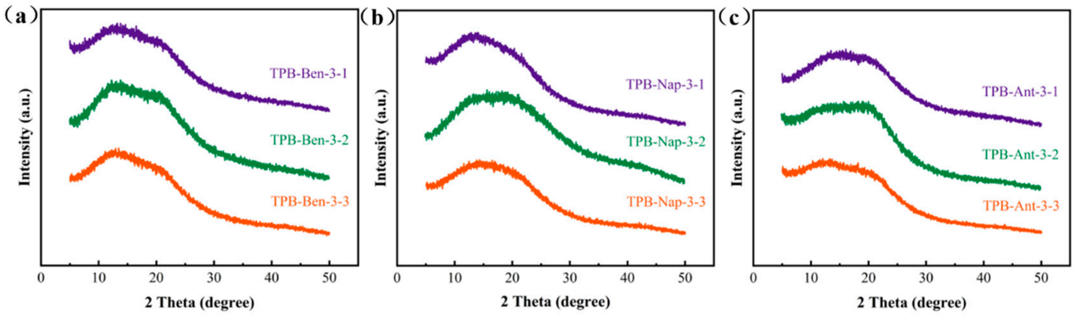

Figure S1. PXRD pattern of (a) TPB-Ben-3-2 (b)TPB-Nap-3-2 (c) TPB-Ant-3-2.

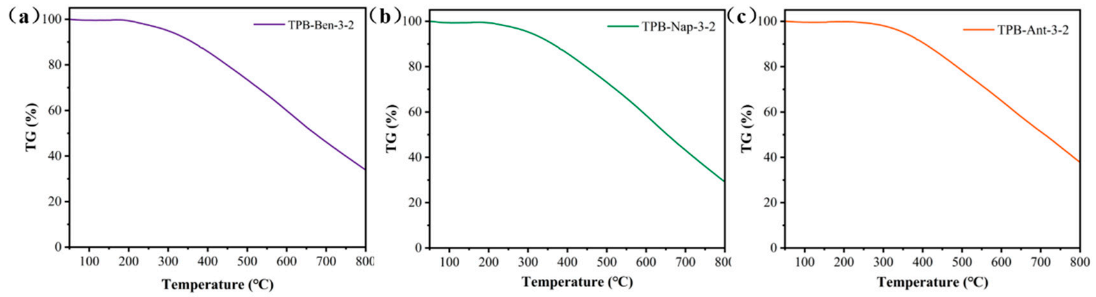

Figure S2. The TGA curves of (a) TPB-Ben-3-2 (b)TPB-Nap-3-2 (c) TPB-Ant-3-2.

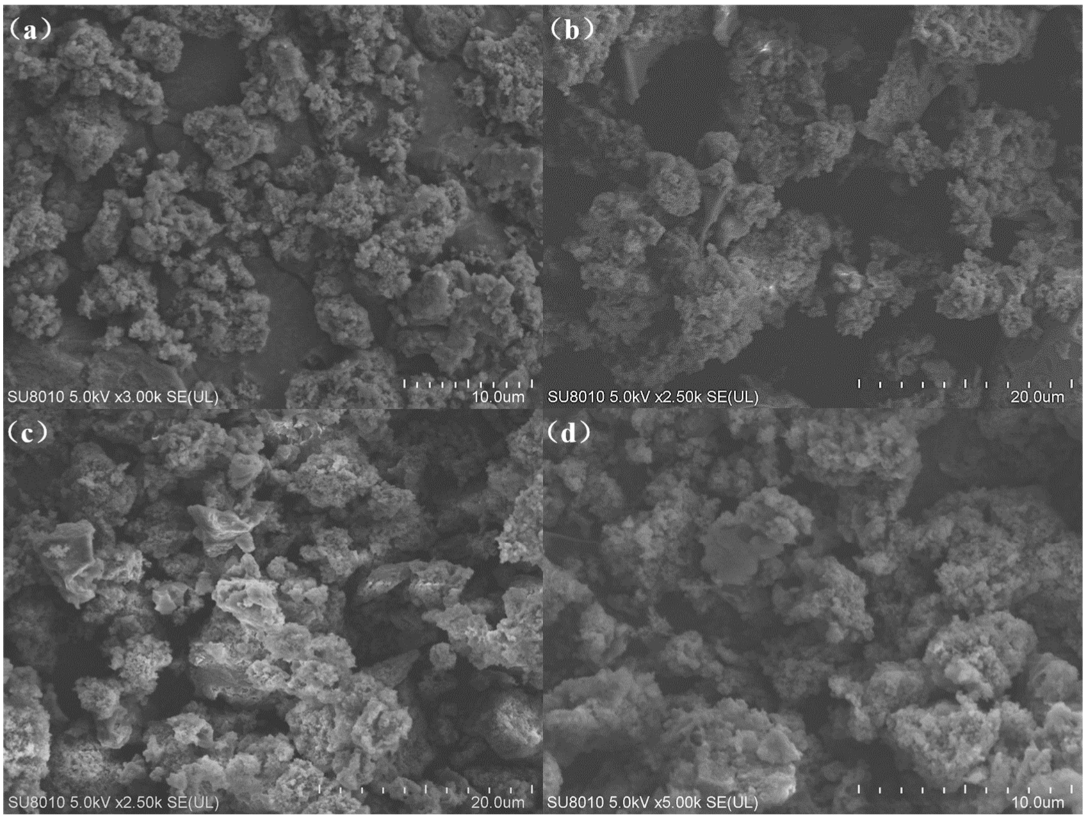

Figure S3. SEM images of (a)TPB-Ben-3-2,(b) TPB-Nap-3-2,(c,d)TPB-Ant-3-2.

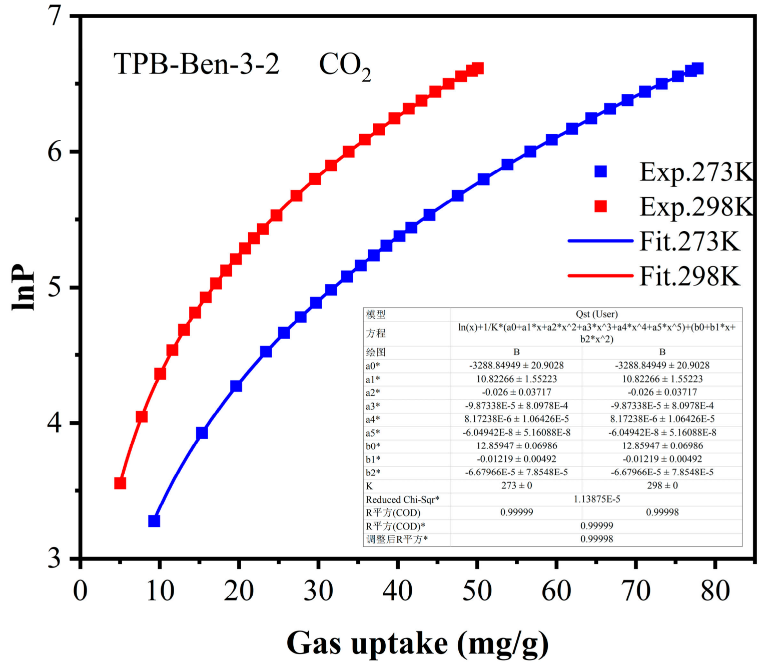

Figure S4. The virial fitting of CO_2 sorption data for TPB-Ben-3-2.

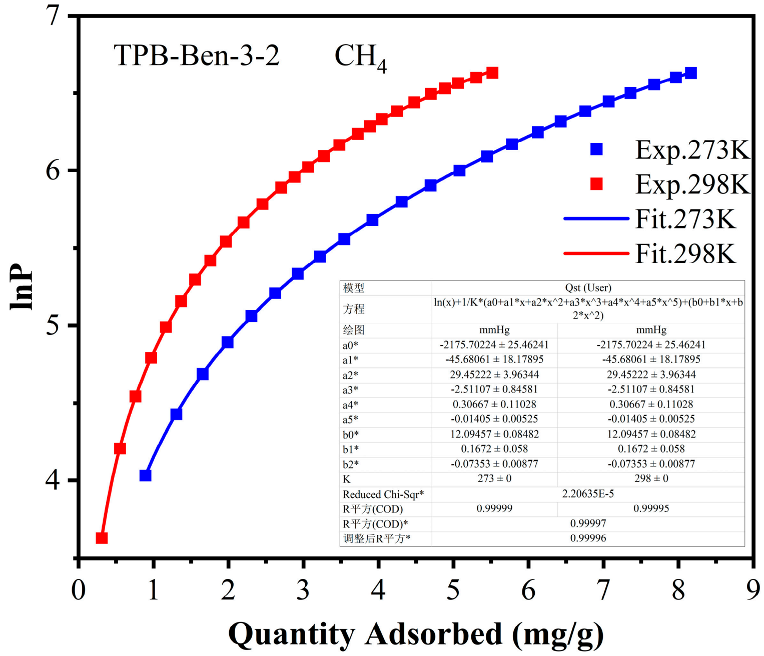

Figure S5. The virial fitting of CH_4 sorption data for TPB-Ben-3-2.

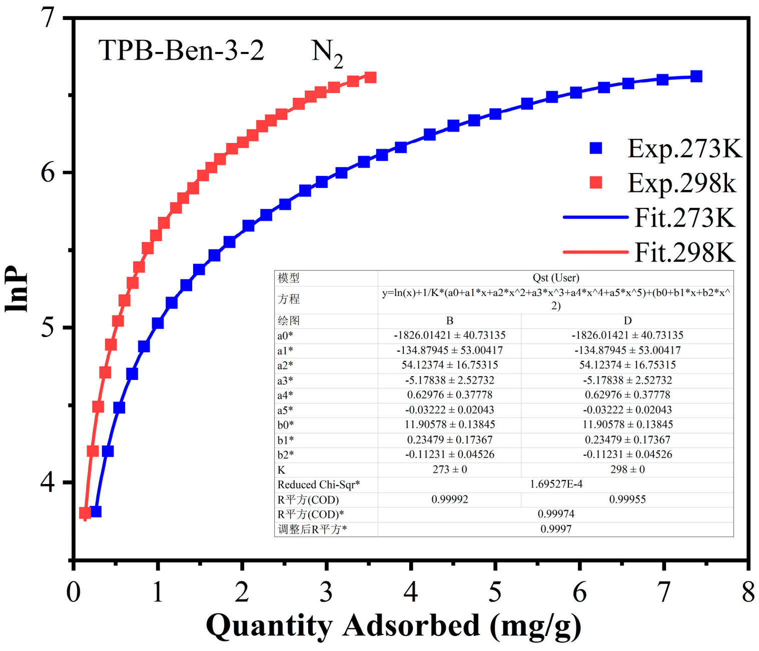

Figure S6. The virial fitting of N_2 sorption data for TPB-Ben-3-2.

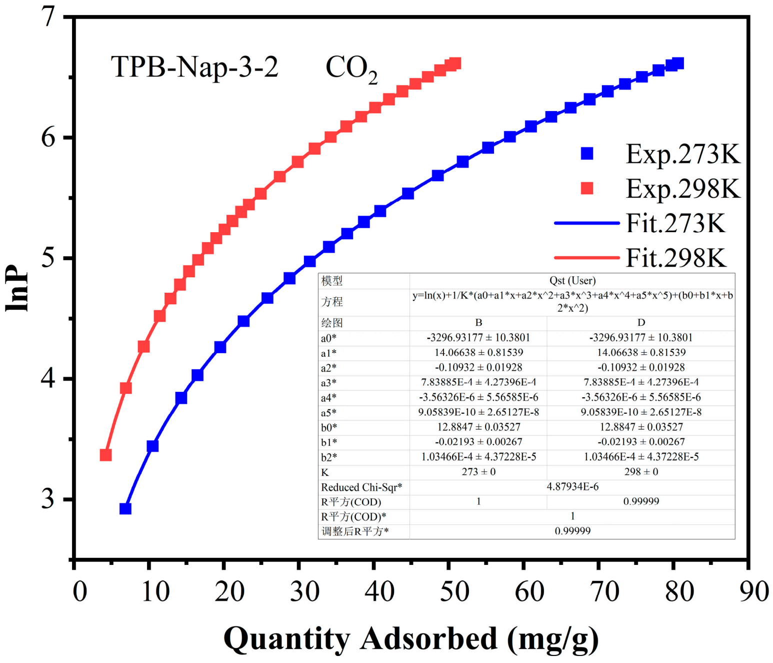

Figure S7. The virial fitting of CO_2 sorption data for TPB-Nap-3-2.

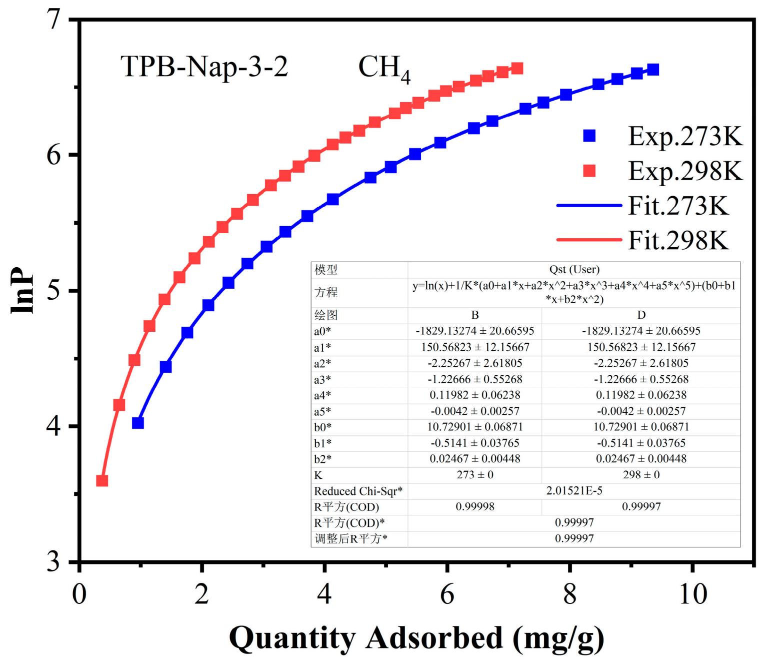

Figure S8. The virial fitting of CH_4 sorption data for TPB-Nap-3-2.

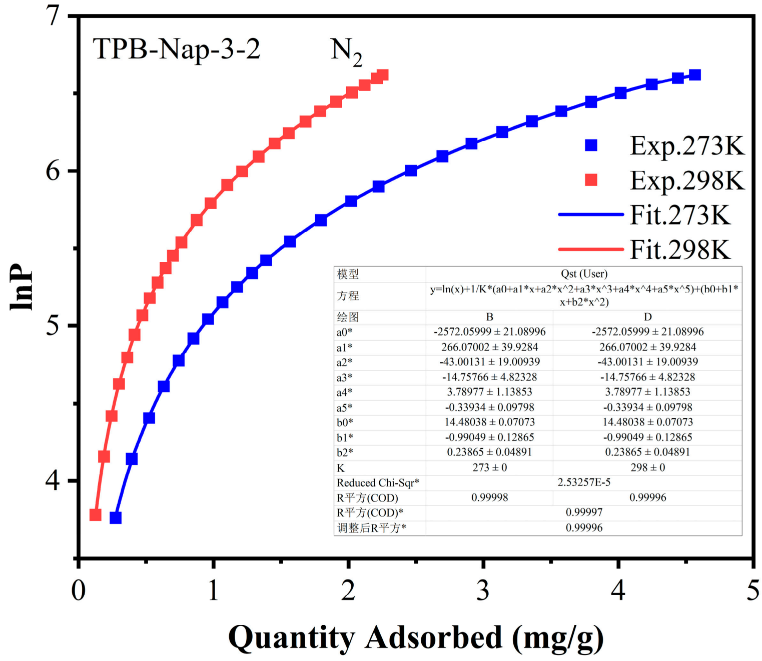

Figure S9. The virial fitting of N_2 sorption data for TPB-Nap-3-2.

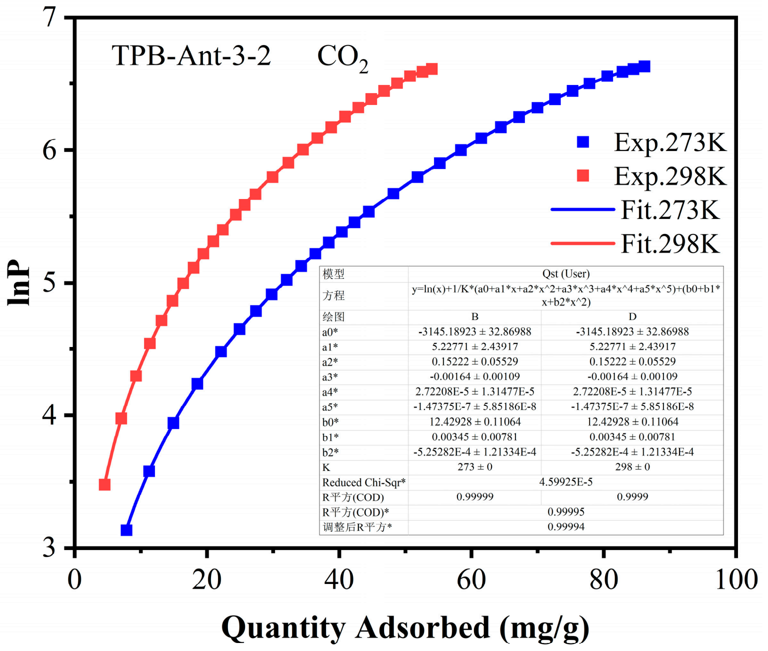

Figure S10. The virial fitting of CO_2 sorption data for TPB-Ant-3-2.

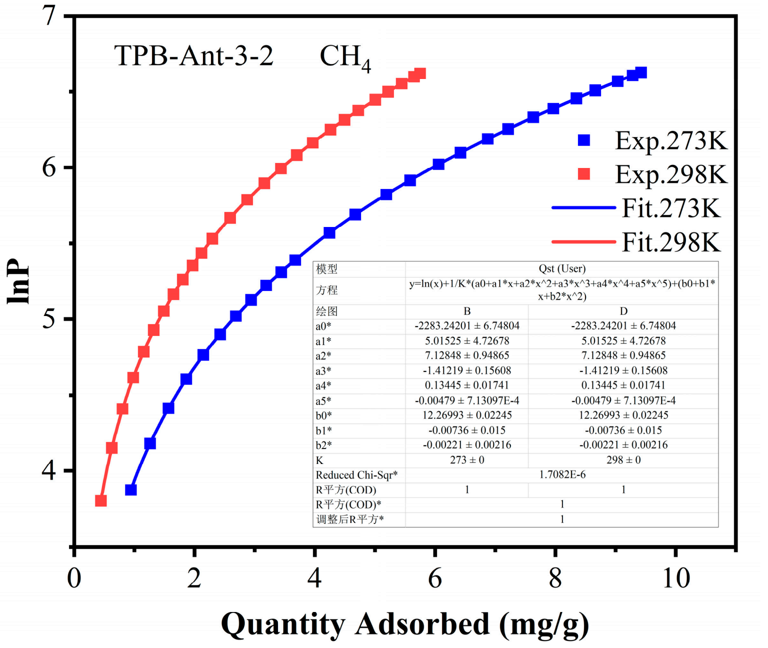

Figure S11. The virial fitting of CH_4 sorption data for TPB-Ant-3-2.

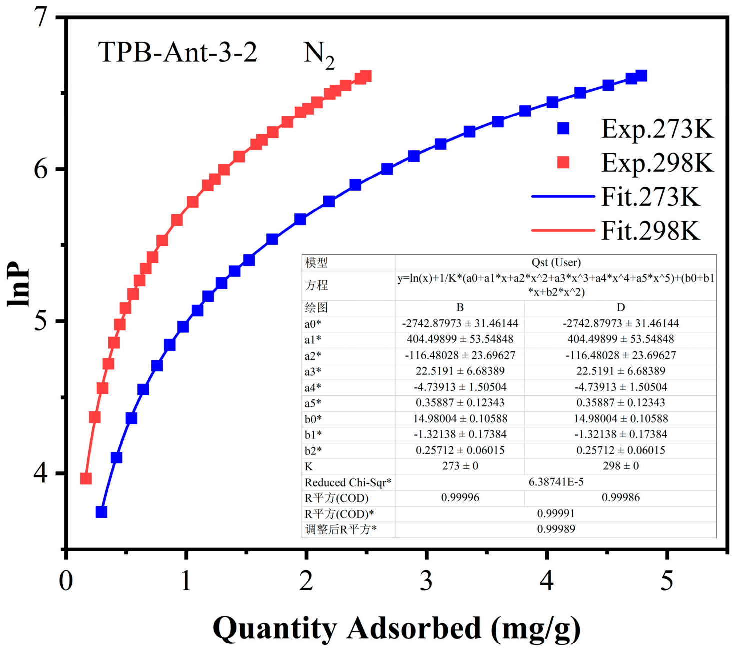

Figure S12. The virial fitting of N_2 sorption data for TPB-Ant-3-2.

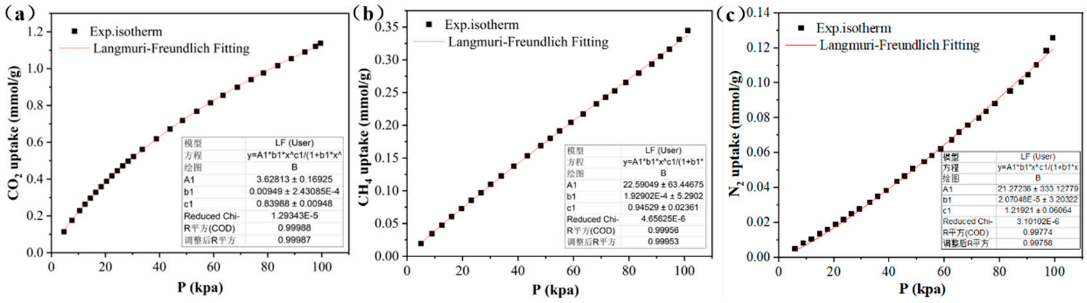

Figure S13. CO_2, CH_4, and N_2 adsorption data of TPB-Ben-3-2 fitted by the dual site Langmuir Freundlich model at 298 K.

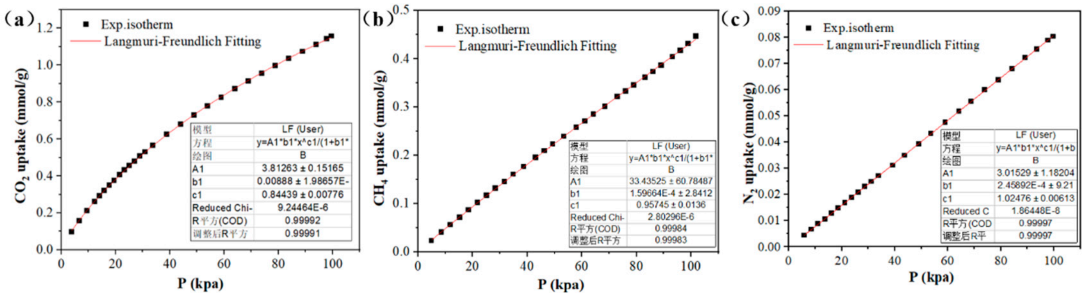

Figure S14. CO_2, CH_4, and N_2 adsorption data of TPB-Nap-3-2 fitted by the dual site Langmuir Freundlich model at 298 K.

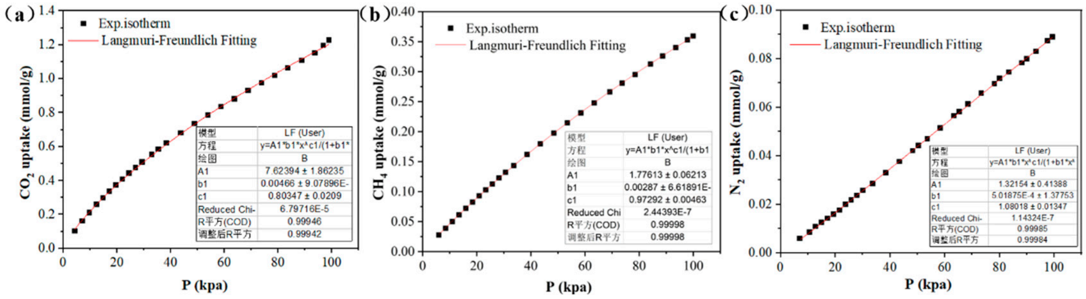

Figure S15. CO_2, CH_4, and N_2 adsorption data of TPB-Ant-3-2 fitted by the dual site Langmuir Freundlich model at 298 K.

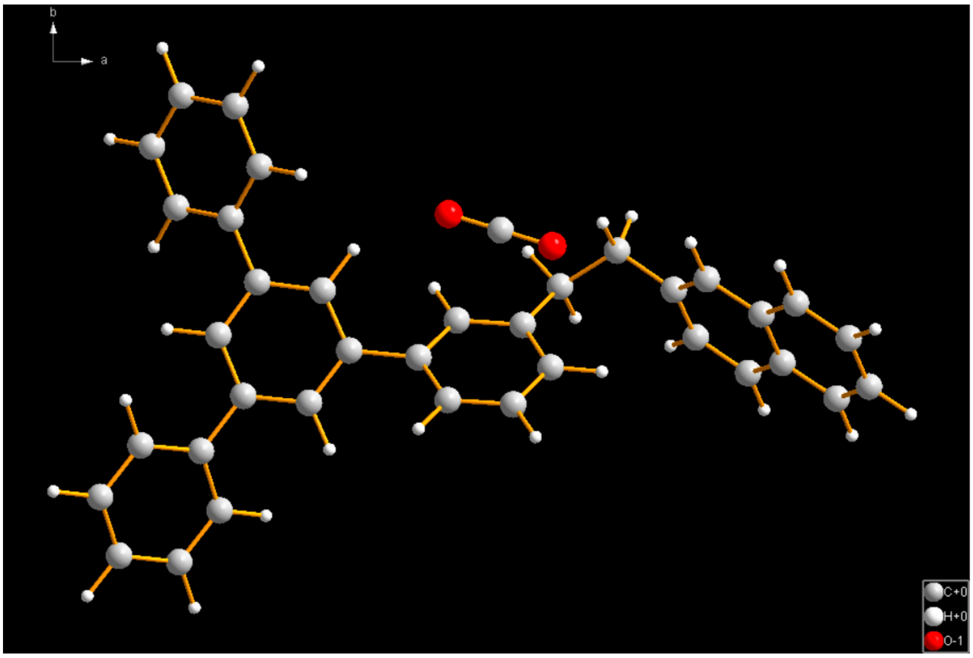

Figure S16 The optimal binding sites of CO_2 was calculated by density functional theory (O, red; C, gray).

3. Supplement Tables

Table S1. CO_2 adsorption capacity and Q_{st} values comparison with reported porous materials.

Reported porous materials	CO_2 adsorption capacity（298 K）（mmol/g）	Q_{st}
PCN-222	1.16	18.3
UPC-70	0.54	17.7
LCU-105	5.36	19.2
LCU-106	4.02	20.6

MIP-202	0.56	30.7
SAPO-17	1.72	38.8
SMOF-SIFSIX-1	1.5	43.98
UTSA-49	3.08	27
ZJU-197	1.20	24.1
TPB-Nap-3-2	1.27	23.4
IITKGP-12	2.004	35.2
Qc-5-Cu	2.16	36
CoIPA	0.82	30.2

Reference

(1) Gómez-Gualdrón, D. A.; Moghadam, P. Z.; Hupp, J. T.; Farha, O. K.; Snurr, R. Q. Application of consistency criteria to calculate BET areas of micro- and mesoporous Metal–Organic Frameworks. *J. Am. Chem. Soc.* **2015**, *138*, 215-224.

(2) Amankwah, K. A. G.; Schwarz, J. A. A modified approach for estimating pseudo-vapor pressures in the application of the Dubinin-Astakhov equation. *Carbon.* **1995**, *33*, 1313-1319.

(3) Chen, K.-J.; Madden, D. G.; Mukherjee, S.; Pham, T.; Forrest, K. A.; Kumar, A.; Space, B.; Kong, J.; Zhang, Q.-Y.; Zaworotko, M. J. Synergistic sorbent separation for one-step ethylene purification from a four-component mixture. *Science.* **2019**, *366*, 241-246.

(4) Myers, A. L.; Prausnitz, J. M. Thermodynamics of mixed-gas adsorption. *AIChe J.* **1965**, *11*, 121-127

(5) Yoon, J. W.; Chang, H.; Lee, S.-J.; Hwang, Y. K.; Hong, D.-Y.; Lee, S.-K.; Lee, J. S.; Jang, S.; Yoon, T.-U.; Kwac, K.; et al. Selec-tive nitrogen capture by porous hybrid materials containing accessible transition metal ion sites. *Nat. Mater*. **2016**, *16*, 526-531.
